# A Survey of Overlooked Viral Infections in Biological Experiment Systems

**DOI:** 10.1371/journal.pone.0105348

**Published:** 2014-08-21

**Authors:** Yajing Wang, Hui Wang, Kunhan Xu, Peixiang Ni, Huan Zhang, Jinmin Ma, Huanming Yang, Feng Xu

**Affiliations:** 1 College of Biotechnology, Tianjin University of Science and Technology, Tianjin, China; 2 College of Pharmacy, State Key Laboratory of Medicinal Chemical Biology and Tianjin Key Laboratory of Molecular Drug Research, Nankai University, Tianjin, China; 3 NERC/Centre for Ecology and Hydrology, Wallingford, Oxfordshire, United Kingdom; 4 Beijing Genome Institute (BGI), Yantian District, Shenzhen, China; 5 Department of Zoology, University of Oxford, Oxford, United Kingdom; 6 BGI-Tianjin, Airport Economic Area, Tianjin, China; 7 Tianjin International Joint Academy of Biotechnology and Medicine, Tianjin, China; 8 Prince Aljawhra Center of Excellence in Research of Hereditary Disorders, King Abdulaziz University, Jeddah, Saudi Arabia; 9 James D. Watson Institute of Genome Science, Hangzhou, China; CNRS UMR7622 & University Paris 6 Pierre-et-Marie-Curie, France

## Abstract

It is commonly accepted that there are many unknown viruses on the planet. For the known viruses, do we know their prevalence, even in our experimental systems? Here we report a virus survey using recently published small (s)RNA sequencing datasets. The sRNA reads were assembled and contigs were screened for virus homologues against the NCBI nucleotide (nt) database using the BLASTn program. To our surprise, approximately 30% (28 out of 94) of publications had highly scored viral sequences in their datasets. Among them, only two publications reported virus infections. Though viral vectors were used in some of the publications, virus sequences without any identifiable source appeared in more than 20 publications. By determining the distributions of viral reads and the antiviral RNA interference (RNAi) pathways using the sRNA profiles, we showed evidence that many of the viruses identified were indeed infecting and generated host RNAi responses. As virus infections affect many aspects of host molecular biology and metabolism, the presence and impact of viruses needs to be actively investigated in experimental systems.

## Introduction

Viruses infect all prokaryotic and eukaryotic species and are one of the major sources of disease-causing agents. However, our knowledge of the world’s virosphere and viromes in ecosystems is still very limited [1]–[3]. In addition to the knowledge gap on unknown viruses, unexpected infections/contaminations made by known viruses are not rare. Effective detection of infections by unexpected viruses still poses a significant technical challenge, not only for environmental samples but also for laboratory specimen and even reagents (e.g., [4]–[13]). It is rather important to consider the effects of unexpected virus infections in biological experiments designed for controlled conditions, because virus infections generate host antiviral immune responses that consume energy and often affect host metabolism and development. An assessment on virus infections in experimental systems is therefore necessary.

New technologies, particularly those associated with Next Generation Sequencing (NGS), now offer powerful tools to detect the presence of viruses in any biological samples. For example, metagenomics protocols have been successful on making virus survey and discovery in many case studies [14]–[18]. These techniques can detect the presence of a virus without the requirement of prior suspicion. However, the presence of a virus does not always equate to an infection with a biological impact. As a viral infection normally triggers host immunological responses against the infection, detection of an anti-viral immunity is used as an indicator of a genuine infection, e.g. host specific antibodies against animal virus infections. It has been suggested that eukaryotic cells may employ the RNA interference (RNAi) mechanism to against viral infections. RNAi, also known as post-transcriptional gene silencing (PTGS), is mediated by small interfering RNAs (siRNA) [19]–[21]. In the infected cells, the animal ribonuclease Dicer and plant Dicer-like (DCL) enzymes catalyse viral RNAs with double-stranded structures, producing virus-derived small interfering RNAs (vsiRNA). The vsiRNAs are incorporated into the RNA-induced silencing complex (RISC) by the Argonaute (Ago) proteins, which use the vsiRNAs as guiding strands to search for the RISC targets, single stranded viral RNAs (e.g. mRNAs), by complementary homology. The vsiRNAs and other virus-derived small RNAs (vsRNA) can be cloned and sequenced (e.g., [22]). Because different Dicers and DCLs produce vsiRNAs with certain lengths, Dicer/DCL pathways triggered against viral infections can be determined by using vsRNA length distributions [23]. Animal RNAi also involves interacting piRNAs (piwi-interacting RNA) and rasiRNAs (repeat associated small interfering RNA) that interact with the PIWI protein and are generally larger than the Dicer/DCL products [19]. Prokaryotic cells have the CRISPR (Clustered Regularly Interspaced Short Palindromic Repeats) system to protect against exogenous genetic elements [24], [25]. All sRNAs produced by Dicer/DCL, PIWI and CRISPR systems can be detected by NGS protocols.

To assess the presence of viruses and infections in experimental systems, we used sRNA datasets of 94 recently published papers of 10 model species to search for known viruses by homology based BLAST (Basic Local Alignment Search Tool) program [26]. Depending on the conserved BLASTn screening, sequences of at least 1 virus were detected from datasets of 28 publications. Two publications reported the virus infections and all reported viruses were detected from the corresponding datasets. Length distributions of vsRNA were obtained for each virus fragment detected in each dataset. Based on the vsRNA profiles, we were able to determine infections in the reported experiments. Possibilities of viral contaminations were discussed.

## Materials and Methods

### Small RNA Dataset Extraction and Process

To identify and download sRNA sequence libraries, we used the advanced search function (http://www.ncbi.nlm.nih.gov/gds/advanced) of the NCBI [27] Gene Expression Omnibus (GEO) (http://www.nchi.nlm.nib.gov/geo) Database (Figure S1). The small RNA high-throughput sequencing experimental series used in this study were from the model species of *Homo sapiens*, *Mus musculus*, *Danio rerio*, *Drosophila melanogaster*, *Caenorhabditis elegans*, *Arabidopsis thaliana*, *Glycine max*, *Oryza sativa*, *Triticum aestivum* and *Zea mays*. The datasets were identified using query key words ((((small RNA) OR short RNA) OR sRNA) AND “high throughput sequencing” [Platform Technology Type]) AND model organism [Organism]. Only files with size <2 GB were downloaded and analysed. All datasets used were associated with papers published before 17 April, 2013. The NCBI accession numbers of all data libraries used are provided in supplementary files (Table S1). The sequence files were converted to the Fasta format of 17–36 nt long reads. Removal of tRNAs, rRNAs, snRNA, and snoRNA [28] was performed by filtering the read sequences using the fRNAdb website (ver. 3.4, http://www.ncrna.org/frnadb/), a database for comprehensive non-coding RNA sequences [29]. Two sets of the sequence data (redundant and non-redundant) were made available for each library and used appropriately in different analyses.

### Small RNA *De novo* Assembly

Small RNA sequences from each individual library were assembled using the SOAPdenovo-trans (SOAPdenovo-Trans-31 kmer, ver.1.0, http://soap.genomics.org.cn/SOAPdenovo-Trans.html) and Velvet [30] (ver.1.2.07, http://www.ebi.ac.uk/~zerbino/velvet/) programs with different series of kmer parameters (k = 15, 17, 19, 21, 23). The outputs from SOAPdenovo-trans and Velvet assembly were re-assembled once again using the Velvet *de novo* assembly program, using combined kmer series (k = 39, 41, 43, 45, 47, 49). The assembled contigs (≥50 nt in length) were extracted using an in-house Perl script. Finally, contigs were filtered for redundancy using the CD-HIT program [31] (ver.4.5.4, https://code.google.com/p/cdhit/downloads/list).

### Mapping Reads to Assembled Contigs

To validate the assembled contigs, the Bowtie 2 program [32] (ver. 2.1.0, http://bowtie -bio.source-forge.net/bowtie2/index.shtml) was used to map all reads back to each contig with parameters as “-N 0 -L 16 -i S,1,0.75–local -a -I 16”. The output file was analyzed by the Samtools software package [33] (ver.0.1.7, http://samtools.sourceforge.net), the BEDtools [34] (ver.2.17.0, http://code.google.com/p/bedtools) program and in-house Perl scripts to calculate the read coverage of each contigs. Only contigs with >95% read coverage were used for further analyses.

### Homology Based Search for Viral Sequences

The assembled contigs were used as query sequences to screen against a local copy of the NCBI nucleotide (nt) database (ftp://ftp.ncbi.nlm.nih.gov/blast/db/FASTA/) using the BLASTn program [26] with an e-value cutoff threshold of e-5. The xml format outputs of BLASTn were screened for virus hits by an in-house Perl script with criteria of (i) ≥80% identity to a known viral sequence and (ii) ≥95% of the contig length was matched to the subject viral sequence. Positions of the viral contigs on each virus genome were plotted using R 2.15.1 program.

### Analyses of Antiviral sRNA Profile

Virus reads were extracted by mapping all sequence reads (100% identity without mismatch) to the detected viral contigs using Bowtie2, Samtools and BEDtools programs. Read counts were converted to counts per million (CPM). Size distribution of virus-specific reads of each overlooked virus from each sample library was calculated using Perl scripts and represented into heatmap using R 2.15.1 program. This analysis was used to identify the RNAi pathways responsible for the production of the identified vsiRNAs. To determine pathway variation, χ^2^ tests were performed using MiniTab-16.

### Mapping vsRNA Reads to Each Virus Genome

To support the results of vsRNA profile, the Bowtie 2 program was used to map all reads of each sample to the virus genomes without any mismatch. The output file was processed by in-house Perl scripts and the R 2.15.1 program to display the vsRNA positions on virus genomes.

## Results

### Small RNA library, contig assembly and BLAST search for virus homologue

**Figure 1:**
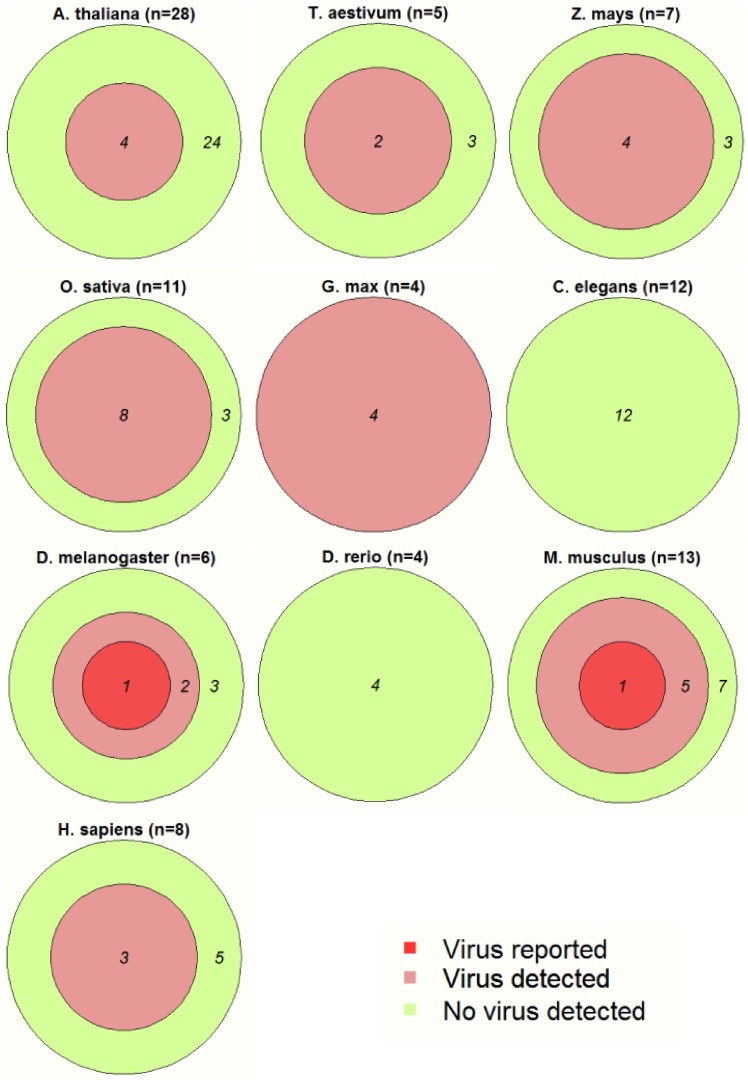
GEO libraries containing viral sequences. Nested sets represent numbers of articles (Table S1) with Virus Reported, Virus Detected and No Virus Detected for each host species examined in this study.

**Figure 2:**
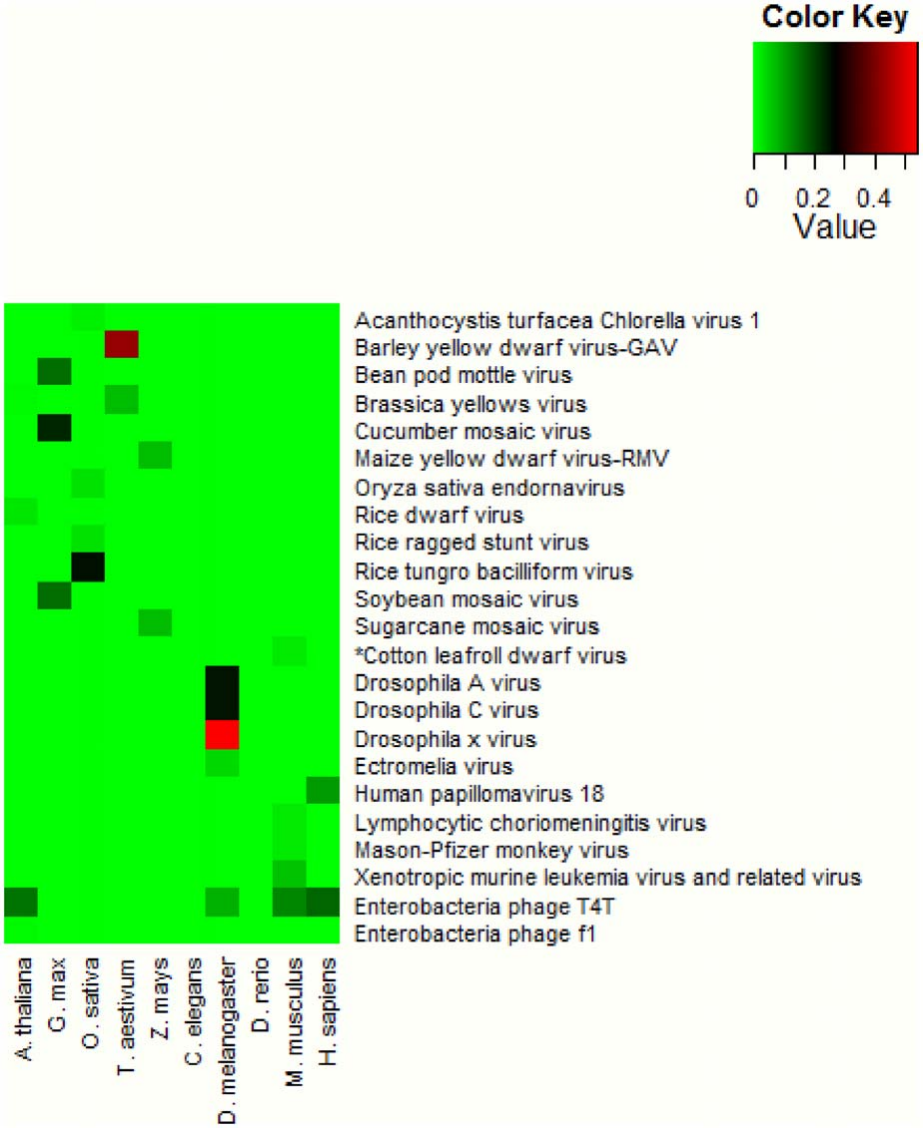
Heat map of viruses detected in each organism. The virus detection rate (DR) was calculated for each virus in each host species using the positive sample number divided by the total number. An asterisk is used to mark the only animal sample (M. musculus, GSM947964) that was positive for a plant virus (*Cotton leafroll dwarf virus*).

A total of 517 sRNA libraries associated with 94 recent publications (Table S1) of 10 model species (Figure 1) were downloaded from the NCBI Gene Expression Omnibus (GEO, http://www.nchi.nlm.nib.gov/geo) database. The short reads were assembled for each library and in total, 4,195,253 contigs ≥50 nt were obtained. To avoid possible assembly artefacts, only contigs with greater than 95% coverage by the original sequence reads were used in this work. The contigs were screened against the NCBI NT database using the Standard Nucleotide BLAST (BLASTn) program. If a contig had the highest BLAST score against a viral sequence with a minimum 80% identify and 95% of the contig length was matched to the subject viral sequence, the contig was deemed as an identifiable virus hit. In total, 461 contigs (Table 1 and Text S1, fasta file of the viral contigs) from 23 overlooked viruses were identified in 8 out of the 10 model species used (Figure 2 and Table S1). In the animal species, the majority of the overlooked viruses were from cell lines (Table 1). Each viral contig was mapped onto the NCBI reference genome of the appropriate virus (Table S2 and Figure S2). Approximately 30% (28 out of 94) of the publications investigated (Table S1) contained at least 1 virus contig in their associated libraries. Two publications described virus infections by the *Murid herpesvirus*, *Rift Valley Fever virus* and *Vaccinia virus* in the libraries we used. All of these viruses were reported by the BLASTn search using assembled contigs (Figure 1 and Table S1). The use of experimental vectors containing viral components was reported in 20 publications (Figure 1 and Table S1), providing a possible origin for some of the viral sequences detected. However, in the majority of the publications in which virus infections were detected, there was no identifiable source of the non-vector viral sequences (Figure 1 and Table S1).

**Table 1:** An overview of overlooked viruses in published sRNA libraries used in this study.

Classification	ModelOrganism	Number ofGEO Library(Cell line)	Library withOverlooked Viruses(Cell line)	OverlookedVirus	TotalContigs	ViralContigs
Plant	A. thaliana	182	32	5	1,167,645	145
	G. max	13	4	3	16,335	88
	O. sativa	63	17	4	300,900	59
	T. aestivum	14	5	2	52,576	57
	Z. mays	14	2	2	257,357	9
Invertebrate	C. elegans	87	0	0	416,472	0
	D. melanogaster	24(15)	15(13)	5	141,598	57
Vertebrate	D. rerio	36	0	0	641,055	0
	M. musculus	47(15)	11(7)	4	315,420	25
	H. sapiens	37(20)	10(10)	2	33,122	21
	Total	517(50)	96(30)	23	3,342,480	461

### sRNA mapping

**Figure 3:**
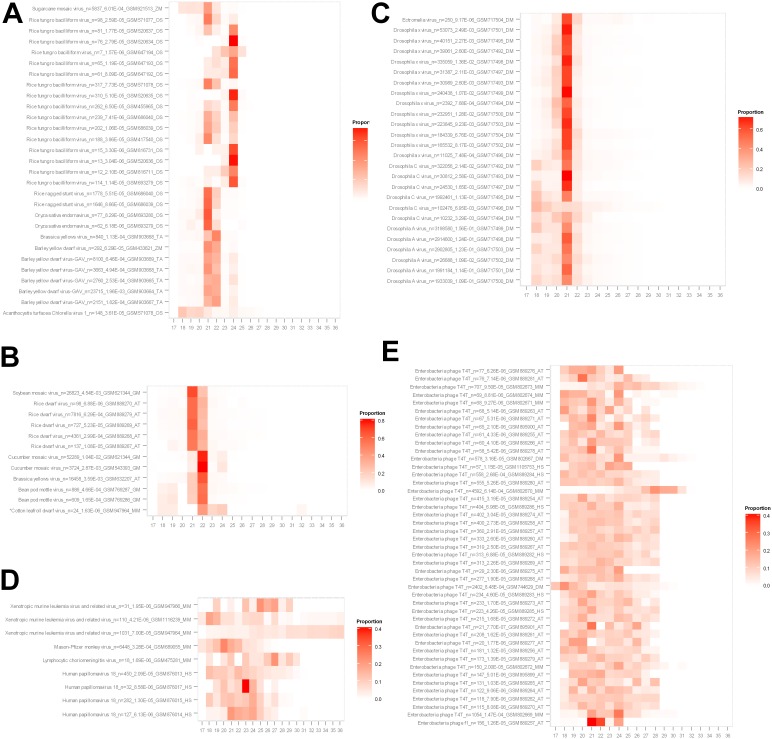
Length distributions of sRNAs matched to the virus contig sequences. Heat maps show the proportions of vsRNAs with certain length (X-axis: 17–36 nt, Y-axis: virus name_read count_abundance in CPM_dataset_host abbreviations. AT: *A. thaliana*, DM: *D. melanogaster*, GM: *G. max*, HS: *H. sapiens*, MM: *M. musculus*, OS: *O. sativa*, TA: *T. aestivum* and ZM: *Z. mays*). Panel A: Monocot host species (TA, ZM, OS); Panel B: Dicot host species (AT, GM, An asterisk was used to mark the only animal sample, M. musculus, GSM947964, which was positive of a plant virus, *Cotton leafroll dwarf virus*); Panel C: Invertebrate host species (DM); Panel D: Vertebrate host species (MM, HS); Panel E: Phages in plant species (AT); Panel F: Phages in animal species (DM, MM, HS).

All of the detected virus contigs were mapped by the sequence reads from the original sRNA libraries. Positions and orientations of the mapped virus reads are shown in Table S2 and Figure S2. Furthermore, all vsRNA reads of each sample were mapped to the virus genomes (Figure S3). Multiple vsRNA locations suggested the likelihood of genuine virus presence whereas single location might suggest random contamination. There were some viruses that were not fully covered. These gaps may be due to viral polymorphisms between the detected viruses and the reference sequences used. In some cases, there were possibilities that some viral sequences in the raw data might have been filtered out by the original researchers. Similarly to the lone coverage at a single location, large coverage gaps represented low confidence on genuine viral infection. The occurrence rates (number of positive samples divided by number of total samples) of each virus (according to the BLASTn annotation) were calculated for each host species, and were represented in Figure 2. There were 13 plant viruses detected in 5 plant species and 8 animal viruses in 3 out of 5 animal species. Only one animal sample (*M. musculus*, GSM947964) was positive for a plant virus (*Cotton leafroll dwarf virus*, marked with asterisks in Figure 2, 3B), but all plant samples were negative for animal viruses. Such a reasonable virus-host association suggested that the majority of plant and animal virus sequences detected were not likely due to possible post-sampling contamination, which could occur at random. Meanwhile, 2 Enterobacteriaceae phages were detected in *Arabidopsis* samples (Figure 2), suggesting sequence contaminations that could also be possibly due to samples contaminated by bacteria carrying phage sequences. No viruses were detected in samples of *C. elegans* and *D. rerio*. Due to the nature of homology based screening using BLASTn that detects known viruses, false negatives may occur because of the limitation of known viruses infecting *C. elegans* and *D. rerio* in the NT database.

### vsRNA profile

Length distributions of vsRNA populations were made for each virus species in each sample. The proportions of each length species (17–36 nt) were calculated and are represented in Figure 3. In the plant viruses (Figure 3A & 3B), the vsiRNAs were dominated by 21, 22 and 24-nt species (only one exception for the *Acanthocystis turfacea Chlorella virus 1* which infects Chlorella algae, NCBI Accession Number NC_008724, Figure 3A), indicating that the majority of vsRNAs were plant DCL products that mediated antiviral RNAi silencing (known as PTGS in plants). However, significant differences were observed for the dominance of the 21, 22 and 24-nt vsiRNA species (Chi-Sq = 64103.909, DF = 96, *P* = 0.000, using data of contigs with mapped reads n >100 in Figure 3A and 3B), indicating that different DCL pathways were employed in different virus-plant associations. In the *Rice tungro bacilliform virus* (RTBV) associated with *Oryza sativa*, two vsRNA phenotypes were observed, i.e., 21 & 22-nt domination vs 24-nt domination (Chi-Sq = 915.378, DF = 22, *P* = 0.000, using data of contigs with n ≥50 in Figure 3A). It has been known that RTBV may integrate its DNA into the host genome [35], [36]. The two types of anti-RTBV vsRNA profiles would be explained as the DCL-4 and DCL-2 dominant anti-infection (free virus) immunity [37], and the DCL-3 dominated anti-transposon (integrated DNA) activity [38], suggesting that the observed viral elements might be derived from activities of free infecting virus and/or transcripts of virus fragements incorporated in the host genome.

A domination of the 21-nt vsRNAs was observed for samples of *D. melanogaster* (Figure 3C), indicating activities of the Dicer pathway in insect antiviral RNAi [20], [39]. However, the predominance of possible Dicer products was remarkably reduced in mammalian samples (Figure 3D) when compared to insect and plant samples (Figure 3A–C). Weak domination of 21 and 22 nt vsRNAs was observed in some of the mammalian (*H. sapiens* and *M. musculus*, e.g. Human papillomavirus _GSM876014_HS, Human herpesvirus_GSM889284_HS and Human herpesvirus 1_GSM678422_MM) samples (Figure 3D). This may support the argument that Dicer mediated RNAi is also used by mammals in antiviral immunity [40], [41]. Instead, significant proportions of larger virus derived sRNAs (25–36 nt) were observed in mammalian samples (Figure 3D). This may suggest a possible involvement of the Piwi pathways in mammals as reported in insect antiviral RNAi (Figure 3C) [42]. However, results of analyzing nucleotide biases at 5′- and 3′-end positions of these large visRNAs were not conclusive (data not shown). There was no solid evidence supporting these mammalian vsiRNAs were the products of Dicer and/or Piwi-protein. Unlike for plant and insect, it is not commonly admitted that vertebrate uses RNAi as an antiviral mechanism. VsRNAs ranging from 25–36 nt could also simply be degradation products derived from virus RNAs.

The vsRNA profiles appeared to be even more complicated for the phage contigs (Figure 3E). Most of the anti-phage profiles were not able to be characterized as the Dicer products. Many of them had relatively equal distributions in the range of 17–28 nt, possibly reflecting nonspecific RNA degradations and/or the CRISPR activity from unidentified bacterial hosts [24], [25].

### Surprising virus-host associations

In addition to the phage-plant/mammal associations, there were other unexpected virus-host associations detected in these sRNA libraries. For the plant viruses, monocot-infecting viruses were detected in *Arabidopsis* (Figure 3B) and dicot-infecting viruses detected in monocots (Figure 3A). For example, the *Rice dwarf virus* (RDV) was detected in sRNA libraries associated with *Arabidopsis*. In particular, a few thousand reads were matched to the viral contigs in libraries GSM889279 and GSM889268 (Figure 3B). The vsRNAs were dominated by the 21-nt species followed by 22-nt species, indicating that they were the products of the DCL-4 and DCL-2 pathways, respectively. The detected RDV contigs displayed 96–100% identity to the subject sequences (NC_003767, Table S2), suggesting that the detected sequences belonged to an infecting RDV strain rather than a novel virus. However, possibilities could not be ruled out for an unknown *Arabidopsis* infecting virus that shares a close phylogenetical relationship to RDV. On the other hand, *Turnip yellows virus* (TuYV) sequences were detected in both *Arabidopsis* (Figure 3B) and wheat (*T. aestivum*) (Figure 3A). The vsRNAs displayed similar length distributions, i.e. domination of 22-nt followed by 21-nt, indicating that DCL-2 was employed as the predominant pathway (over the DCL-4 pathway) against TuYV infections. The TuYV contigs associated with *Arabidopsis* were 99.7–100% identical to the subject sequence (NC_003743, Table S2) while the TuYV contigs from *T. aestivum* were 92.2–100% identical to the subject sequence (NC_003743, Table S2, Figures S2 and S3). Again, the possibility of a wheat-infecting TuYV homologue could not be ruled out. It was surprising to find sequence homologues of the Enterobacteria phage T4T in multiple datasets (Figure 3E). This may suggest the usage and/or contamination of materials with phage/bacteria origin in experimental systems. From the vsRNA length distributions, these phage vsRNAs were not produced by Dicer/DCL enzymes, thus direct phage infections in eukaryotic hosts were unlikely.

## Discussion

Metagenomics strategies based on the NGS technology provide powerful tools for making virus discoveries (e.g., [1]–[18]). Among the techniques, the small RNA approach is unique because it detects the presence of virus as well as RNAi immunity that indicates infection at the same time [8], [12], [13], [43]. The siRNA mediated antiviral immunity is suggested as an ancient mechanism in eukaryotes [19]–[21] and thus can be used for detecting a broad range of virus infections. Unexpected virus infections/contaminations have been reported in laboratory plants (e.g., [12]) and cell lines (e.g., [8]). Data reported here showed that overlooked virus infections were not rare and surprisingly high in certain species. Materials used in more than 20 out of 94 publications contained virus sequences from un-identifiable resources (Figure 1 and Table S1). These nucleotide sequences were identical or highly similar to virus sequences available in the public database (Table S2, Figures S2 and S3). Therefore, they are more likely to be sequences of known viruses rather than putative new viruses. The BLASTx program, which compares deduced amino acid sequences, will be more powerful in searching for novel virus sequences than the BLASTn program used in this study. Results from the BLASTn screen have provided a conserved picture of the general lack of awareness of virus infections in biological experimental systems.

This study also demonstrated the feasibility of screening virus infections using the sequencing datasets produced from experiments not designed for virology studies. By assembly of sequence reads followed by standard BLAST screening, virus hits can be readily detected. Although assessing whether or not an unexpected virus infection may affect the quality of an experiment and the interpretation of results must be decided on a case-by-case basis, knowing that there may be a viral factor involved should generally be considered an improvement to the overall experiment. From a virology point of view, screening for viruses will help to extend our knowledge of the virus-host range, and to understand the host antiviral RNAi and PTGS immunity if sRNA libraries are used. Information about the presence of viruses would also be useful in a broader context because changes to the sRNA population may affect host metabolism and development [44], [45].

## Supporting Information

Figure S1 **Flow chart of bioinformatics procedure.**
(TIFF)

Figure S2 **Distribution of viral contigs mapped to the virus genomes.** Each viral genome fragment was shown as a black bar and each viral contig was represented as a green bar.
(TIFF)

Figure S3 **Positional distribution of viral reads showing vsRNA coverage on the virus genome.** Each viral genome fragment was shown as a black bar and the viral reads were represented as red dots.
(TIF)

Table S1 **Small RNA libraries used and viruses detected.**
(XLS)

Table S2 **BLASTn results of viral contigs mapped to the virus reference genomes.**
(XLS)

Text S1 **Viral contig sequences in the Fasta format.**
(XLSX)
