# Supplementary figures and images for: A Survey of Overlooked Viral Infections in Biological Experiment Systems
Source: PLoS One. 2014 Aug 21;9(8):e105348. doi: 10.1371/journal.pone.0105348 (PMC4140767; doi:10.1371/journal.pone.0105348)

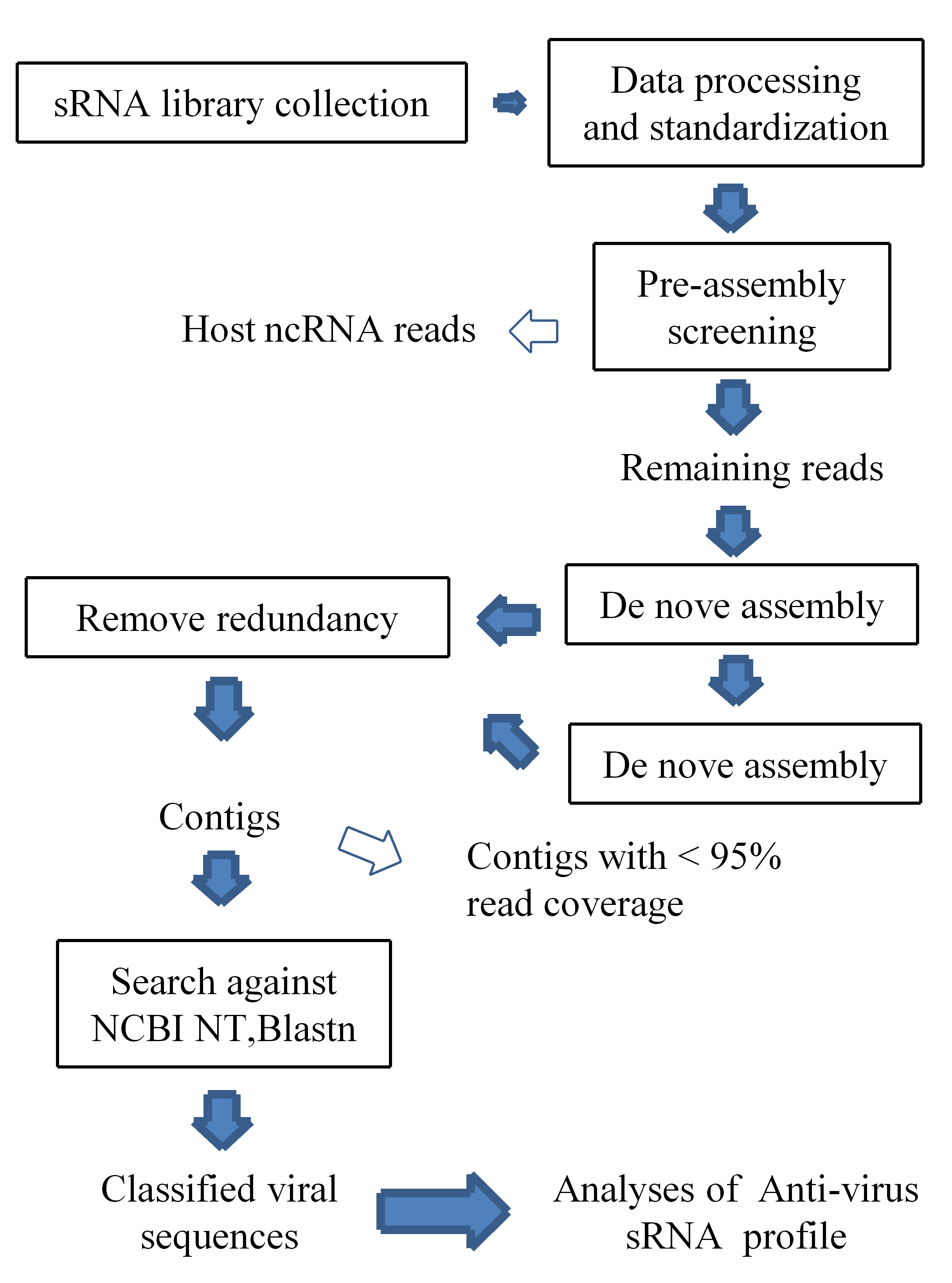

Supplement: Figure S1 — Flow chart of bioinformatics procedure. (TIFF) [file pone.0105348.s001.tiff]

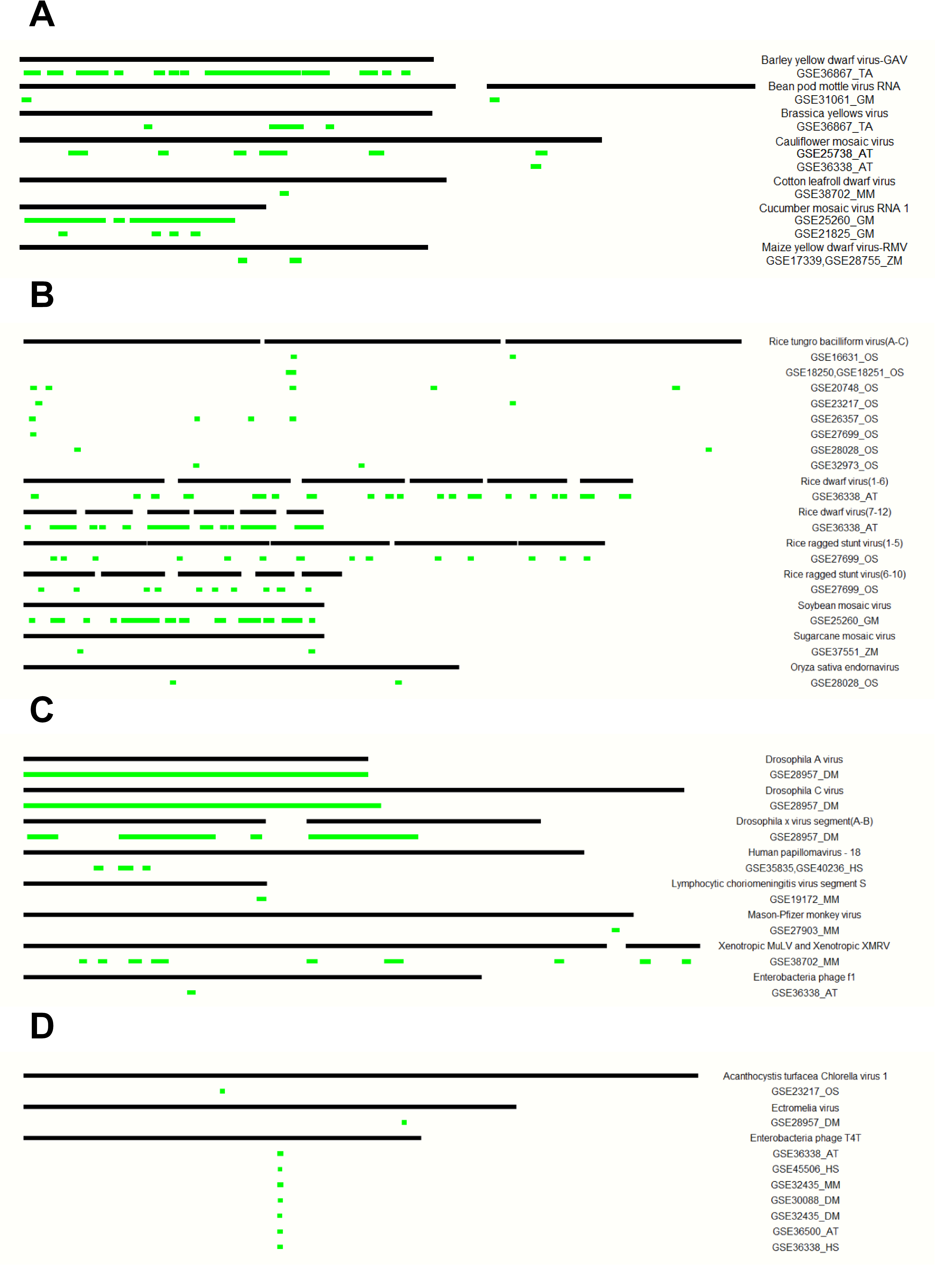

Supplement: Figure S2 — Distribution of viral contigs mapped to the virus genomes. Each viral genome fragment was shown as a black bar and each viral contig was represented as a green bar. (TIFF) [file pone.0105348.s002.tiff]

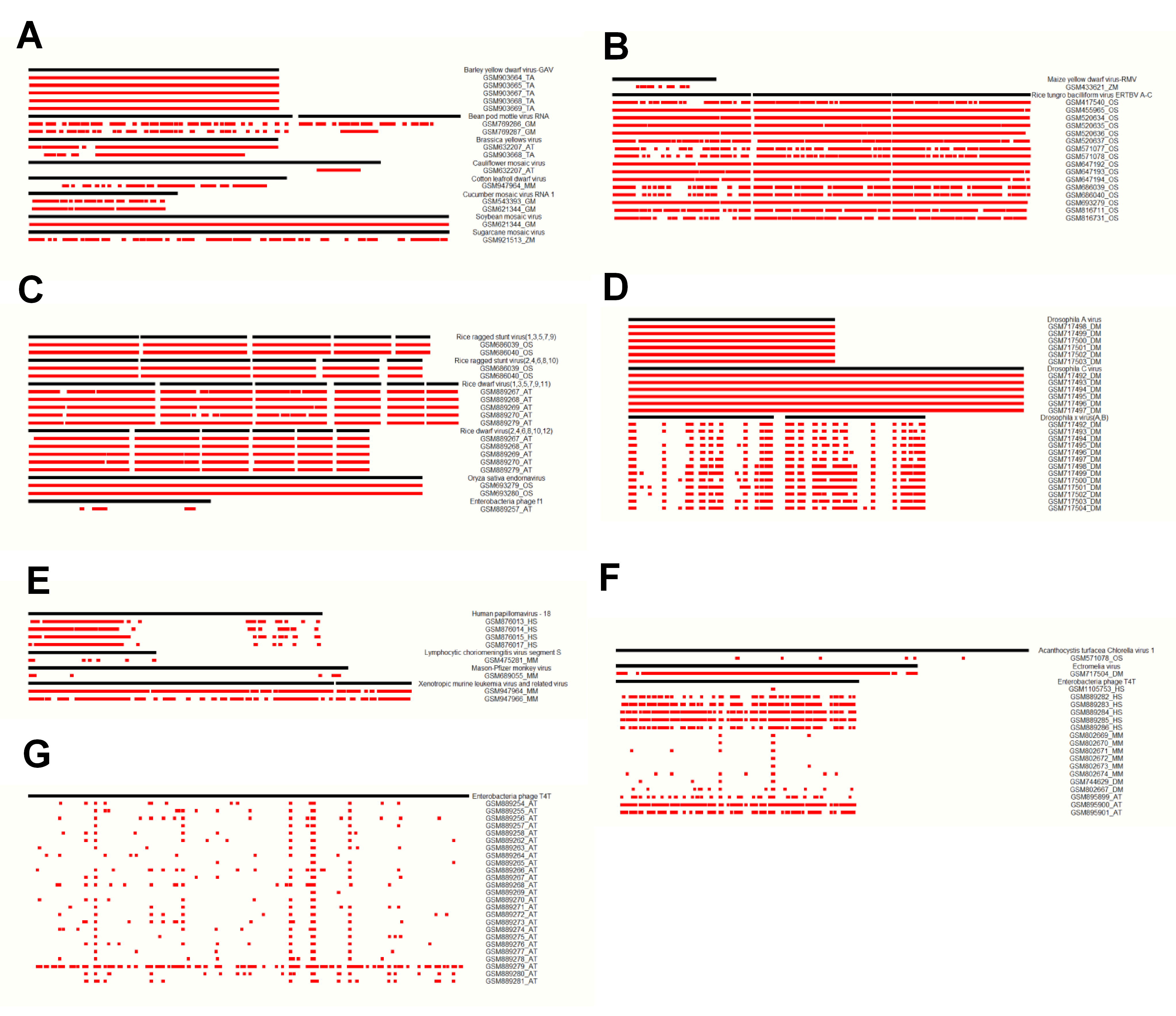

Supplement: Figure S3 — Positional distribution of viral reads showing vsRNA coverage on the virus genome. Each viral genome fragment was shown as a black bar and the viral reads were represented as red dots. (TIF) [file pone.0105348.s003.tif]
